# Emotion Formation in Old Age—The Result of a Top-Down Positive Strategy, General Habituation or a Bottom-Up Decreased Interoception?

**DOI:** 10.3390/brainsci16030261

**Published:** 2026-02-26

**Authors:** Daniela Aisenberg-Shafran

**Affiliations:** Clinical Psychology of Adulthood and Aging Department, and the CMA Center for Multigenerational Psychotherapy and Counseling, Ruppin Academic Center, Emek Hefer 4025000, Israel; danielaa@ruppin.ac.il

**Keywords:** emotion-formation, older-adults, interoception, cognition, habituation, culture

## Abstract

Emotion formation in older adults may be influenced not only by top-down cognitive strategies such as the positivity bias but also by bottom-up physiological changes. Decreased interoceptive awareness in older adults may alter the traditional emotion formation process, potentially reducing the intensity of negative emotions. The frequent physiological changes during the process of aging may lead to habituation that should also be considered as a complementary mechanism. In addition, cultural factors and social norm shifts further complicate emotion formation in aging populations, particularly for immigrants who must navigate unfamiliar social codes while experiencing diminished interoceptive signals. Looking at emotion formation in older adults contributes to the understanding of emotions as a complex construct involving several complementary processes which receive different weights according to the person’s situation.

Emotions in old age are typically found to be more positive; seniors seem to focus less on the negative aspects of experiences. However, the underlying mechanism or changed process of emotion formation should be more carefully examined, considering the physiological changes that occur during aging [1]. In other words, it is not necessarily that older individuals are more positive but rather that emotions are formed based on a top-down emphasis compensating for the decreased awareness of physiological changes that modulate these emotions.

One of the earliest explanations for the nature of emotions is the James–Lange theory [2]. According to this theory, external stimuli provoke physical reactions in us, and the emotions we experience arise from our interpretation of the physical response. In 1927, the Cannon–Bard theory was suggested as an alternative explanation, since people can experience the same physiological reactions with or without actually feeling the same emotions (e.g., heart bit increase during sports activity without the feeling of fear) and since sometimes the realization of emotion is faster than the physiological sweat, for example, that is related to it. Hence, this theory offered the concept that the physical and mental experiences of emotions occur simultaneously and are independent of each other. In 1962, a new theory of emotion emerged, called the two-factor theory—or the Schachter–Singer theory—which combined ideas from the two predecessors [3]. According to this explanation, the physiological response does indeed appear before the emotion, and the same physical phenomena can characterize other and different emotions. An additional cognitive processing stage exists, as information processing provides meaning to our physical sensations. In the first stage, a stimulus creates a physical response. In the second stage, a mental process takes place that examines the situation that led to the feeling and decides which emotion to attribute it to. For example, the surprising sound of a blast in combination with intense light may either be a bomb or the beginning of a surprise party, both leading to an increase in our heart rate. The cognitive process of understanding the context (also by watching others’ responses) will determine whether we feel fear or joy.

As we age, a more positive evaluation of situations is referred to the attentive processing of older adults. This seems in line with the cognitive part of understanding how emotion is created, emphasized in the newer appraisal theories, and first suggested by Arnold and Lazarus [4]. The appraisal theory views emotions as adaptive processes that reflect a complete process of repeated evaluation of the environment that is significant to our well-being. When we are exposed to a stimulus, we engage in an automatic and rapid process of evaluating the nature of the stimulus, its meaning, and its importance for us. This process elicits a physical response, and then an explicit label of emotion. Our evaluation of any event or stimulus we experience will change according to our goals, needs, and values, which may change over time, so that the same stimulus will evoke different emotions on different occasions. This explanation is in line with the positive strategy theory of emotion regulation in aging.

Yet, in all theories, there seems to be an agreement regarding the physiological change that affects emotion, and the discussion is about the strength of the contribution of interpretation and its timing. In the context of aging, I would suggest that we should dwell specifically on the physiological aspect, challenging its role and contribution to the formation of emotion. That is, that greater attention should be given to the ability of interoception—the perception of physical sensations (such as heartbeat, breathing, stomach sensations, and autonomic nervous system activity) that affect an individual’s mood, psychological well-being, and feelings [5]. In other words, when understanding emotion formation, aging provides us with new views. We should examine the weights of the contributing processes, as some changes. In other words, it is not necessarily that older individuals pay attention differently or regulate their emotions more efficiently but perhaps that emotions are experienced differently or should be defined differently. Rather than considering the common process of emotion formation, in old age, the top-down regulation interacts with the fact that older adults may be less aware—due to habituation or loss of sensation—of the physiological changes that modulate emotions [1]. In such cases, emotion processing may rely more on wisdom and practical experience and the need to adjust to changing social norms and behaviors.

Though it is very clear to clinicians that emotions are experienced and often expressed differently in late adulthood, the underlying process is not well-enough explored or understood. Is the process of evoking emotion influenced mainly/partially/equally by a top-down positive strategy, general habituation to frequent physiological changes or a bottom-up decreased interoception?

1. Emotion formation as a top-down positive strategy

Studies have shown a higher motivation to maintain positive affectivity [6] in old age, as well as efficient emotion regulation [7]. The socioemotional selectivity theory (SST), a lifespan theory of motivation [8], suggests that goals change as a function of future time horizons. Hence, older adults are more oriented (top-down attention) to meaningful, self-related positive data, which enhances attention to stimuli that contribute to the formation of positive rather than negative emotions [9,10]. Yet, different studies also found competing results, showing that older adults, compared to young adults, were more attentive to negative expressions [11]. It is important to note that the positive strategy is not the only top-down process that may affect emotion formation, as will be further elaborated later. Nevertheless, it is an important factor in the complementary interaction of processes that form emotion in old age.

2. Emotion formation changes due to habituation to frequent physiological changes

The role of heart rate variability (HRV) in fear learning and extinction has been established [12], underlying several psychotherapy methods for reducing anxiety and obsessive compulsions (for example in cognitive behavioral therapy) [13]. As we age, daily activities may require a physical effort that was not there before. For example, getting out of bed may cause a change in heart rate; additionally, walking quickly to a ringing telephone or to the door with a person waiting outside are expected to increase a person’s heart rate. In addition, medical conditions or response to various medicine may alter HRV. These situations make the change in heart rate a more familiar or frequent event, and in extinction learning the older adult learns that the change in heart rate does not necessarily indicate a threat. Though this alternative is conceptual and does not stand alone regardless of cognitive reinterpretations, it should be considered in the process of emotion formation. There is evidence for amygdala habituation in aging, affecting the emotional response [14]. Thus, heart rate variability gradually makes less of an impact on the decision of event valence. Eventually, in response to a frightening stimulus, the older adult, partially intentionally [15], may not regard the physiological component as being as valuable as before or indicating risk. That is, the process of emotion formation may change, relying less on the physiological factor and more on the cognitive interpretation and reactions of the environment. Interestingly, recent findings also point to the potential of HRV as a tool for measuring stress resilience during aging [16], making it even more relevant for aware observation.

3. Emotion formation affected by decreased interoception

The awareness of the body’s internal sensations—interoception—has been found to change with age. Mendes [17] suggested that a weakened connection between the body and mind dramatically affects the emotional experiences in old age. Older adults are less sensitive to perceptions of physiological changes and reactivity to emotional stimulation is solely reliant on the emotional value of the stimulus and external cues from the environment. Mixed results were reported regarding interoception in old age, viewing the tendency to be internally focused, based on personal beliefs regarding body sensations- introceptive sensibility, and separated from situational components of it. These results comprised the following: *interoceptive sensitivity accuracy*, a considerable objective and measurable measure of detection of internal stimuli [18]; *interoceptive awareness*, the subjective perception of physiological changes in the body. Evidence for age-related decline in both interoception types was reported in recent years [1,14,19]. Even in cases in which interoceptive sensitivity does not alter cognitive functions, the decline did affect emotions. Methods for measuring interoception are problematic, specifically when acknowledging age-related effects. Self-reports of subjective sensations, cardiac-based tasks, and respiration or gastrointestinal sensations are all affected by health conditions and attentive states. The largest intra-variability among the older population compared to any other age group adds to the methodological difficulty. Nevertheless, evidence supports and acknowledges that for older adults there is a decrease in interoception (awareness and accuracy) that mediates emotional experience.

When an older adult does not experience a negative emotion with high intensity, or when negative emotions arise less frequently, we often attribute this to strategies of biasing attention to the positive. However, it is important to analyze the situation more precisely and to understand whether an attentional strategy with compensatory mechanisms that are familiar in old age was indeed activated, or whether a more basic component of the emotion formation—the physiological data, have changed, and therefore the emotional outcome is different. For example, one can imagine that a person who experiences a decrease in interoception (awareness) and does not feel changes in heart rate as before may think that an event of a frightening nature is not so frightening if their heart rate did not increase significantly. Even when there is a physiological change that in the past would have been associated with a frightening interpretation and the arousal of a strong emotion of fear, a current lack of awareness to this change in old age affects the entire emotional algorithm and leads to a less powerful feeling of (negative) emotion. Additional age-related physiological changes may also underly this reduced intensity or negative emotion formation. For example, it was found that older adults show difficulty in disengagement processes, reflected in the insula and cerebellum [20]. Late disengagement from previous stimuli of internal focus may account for lower sense of urgency and slower processing. This itself may decrease the interpretation of body sensations.

It is worth mentioning another influential aspect in old age: **the role of culture in shaping emotions.** Some researchers argue that emotions are the product of a cultural construction, in which they are constructed to fit the demands of the culture in which we live in [21]. Therefore, people will tend to experience emotions that are considered normative in the society in which they live more. When thinking of this cultural effect in older adults, a dissonance may arise as norms change along the years and people sometimes leave their origin culture and move to a new one (immigrants in-land and across countries). The top-down modulation of observing the unfamiliar response of the environment, or the new codes of behavior, affect interpretation and accompanying emotions. Along with decreased interoception to rely upon, a confusing experience may take place. The need for belongingness and normative behavior may often inhibit the common procedure of experiencing emotions to fit society. Decreased interoceptive awareness and lack of understanding local social norms create a unique infrastructure for emotion formation, in which different weights are given to physiological changes and cognitive and environmental evaluations. Here, environmental cues are more likely to receive the highest weight, prolonging the process of evoking emotions, as it is not equally based on inner sensations and perceptions.

In summary, emotion processing in old age is often different than the schemes we use as young adults. Consider, for example, the original study underlying the two-factor theory by Schachter and Singer [3]. Participants received an injection of adrenaline, increasing their heart rate, breathing, sweating and other physical arousal reactions. The rest received a neutral placebo. Only some of the participants received explanations about the effects they would experience as a result of the injection. Then, participants were placed in a room where they met a person who behaved in a way that expressed either joy or anger. Participants who did not know about the connection between the injection and their sensations tended to feel joy or anger depending on the behavior of the person they met in the room. In contrast, participants who received an explanation of the effect of the injection or who received a placebo were less affected by it. Now, think of older participants with lower senses of interoception, or frequent awareness of physiological changes, or a strong fear of thwarted-belongingness and self-ageism. They would probably induce different processing processes, yielding, most certainly, different results. Personal variability affected by personality, wisdom, practical experience, the need to adjust to changing social norms and behaviors, and interoceptive changes in awareness to physiological measures are all contributing factors to what must be understood as a very complex event of emotion. The narrow term of emotion as a reflexive event (even neuropsychologically localized) is simply not enough. The constructionist view, starting with William James [22] through to the modern model [23], seems more reasonable. Psychological aspects contributing to or interfering with the process of emotion formation indicate that it is a multifaceted and both bottom-up and top-down affected process.

As clinicians, it is important to keep in mind that emotions are not automatic by definition but rather complex events, derived from self-experience, personality and age-related wisdom. With personal variability in old age being the greatest of all other age groups, we should curiously examine various potential factors affecting emotion formation of older patients in a different way to the common presentations of known theories. It is recommended to inquire with the patient about the various components that may affect emotion formation in a certain situation, such as awareness to physiological information or changes, pre-conceptions, social norms, interpersonal interactions, personal beliefs, previous experience and expectations. Bearing in mind that chronic condition patients had lower interoceptive accuracy and lower interoceptive sensibility was associated with higher symptom severity or frequency [24], it is also important to pay careful attention to the patients’ condition in order to avoid missing warning signs of somatic or emotional distress in older adults. We should remember that each patient may manifest cognitive–interoceptive–psychological interaction differently, affecting their emotions and behavior. Future studies should focus on adjusted interventions for older adults, to better identify, reflect and personally address emotion processing and its’ self and social implications.
